# Relationship Between Passion and Courage among the Experienced Male Soccer Players

**DOI:** 10.21315/mjms2020.27.4.8

**Published:** 2020-08-19

**Authors:** Erkut Konter, Yee Cheng Kueh, Garry Kuan

**Affiliations:** 1 Dokuz Eylül University Sport Science Faculty, İzmir, Turkey; 2 Unit of Biostatistics and Research Methodology, School of Medical Sciences, Universiti Sains Malaysia, Kubang Kerian, Kelantan, Malaysia; 3 Exercise and Sports Science Programme, School of Health Sciences, Universiti Sains Malaysia, Kubang Kerian, Kelantan, Malaysia; 4 Department of Life Sciences, Brunel University London, United Kingdom

**Keywords:** courage, harmonious passion, obsessive passion, performance, soccer

## Abstract

**Background:**

The nature of the relationship between passion and courage and its influence on soccer performance has yet to be revealed. Thus, the purpose of this study was to examine passion attributes (i.e. harmonious and obsessive inclinations) among male soccer players in relation to the level of courageous characteristics (i.e. mastery, determination, assertiveness, venturesome and sacrificial behaviour), other demographic and player characteristics.

**Methods:**

Data were collected from 278 male soccer players aged 13–38 years (mean [M] = 17.42 ± 4.36) with the number of competitive soccer experiences ranging from 1–28 years (M = 7.51 ± 4.23 years). Participants had at least a year of experience in the sport of soccer completed the validated passion scale, sports courage scale and demographic form.

**Results:**

Analyses revealed that soccer players with higher levels of total courage (*P* < 0.001), have more experience in soccer (*P* = 0.011), and their soccer level being professional (*P* < 0.001) had a significantly higher score in harmonious passion. There was no significant difference in obsessive passion among different level of total courage (*P* = 0.154). However, soccer players with more experience (*P* = 0.011) and higher soccer level being professional (*P* < 0.001) demonstrated a significant higher score in obsessive passion.

**Conclusion:**

In conclusion, soccer players with higher harmonious and obsessive passionate attributes had higher courage (except for mastery). In addition, the courageous and passionate traits of the soccer players played meaningful roles in indicating individual and performance variables.

## Introduction

Players who enjoy playing soccer are autonomously and passionately involved in the game. Many of the world’s best soccer players, for example, Diago Maradona and Cristiano Ronaldo, anecdotally refer to the joy and pleasures they attained from the sport (1). This sense of joy and pleasure could be linked with the self-determination theory by Deci and Ryan (2, 3). This theory is based on the three basic needs of human beings: autonomy (i.e. the need to have control over one’s actions), relatedness (i.e. the need to feel connected with other people) and competence (i.e. the need to have a positive effect on outcomes and surroundings) (1, 4).

Jordet (1) concluded that there is indirect evidence of self-determined involvement in the game that has been cultivated as the foundation within the players who make it into the professional ranks in soccer. Self-determined behaviour that is driven from being intrinsically involved in soccer seems to influence the performance outcome positively. Thus, the self-determined behaviour observed in the soccer players are linked with commitment and passioned.

Vallerand (5) originally developed the passion scale (PS) based on the dualistic passion model (DPM). Passion refers to strong inclination towards an activity that a person likes or loves and finds important, in which significant time and energy are invested (5). According to Vallerand (5), passion can be divided into harmonious passion (HP) and obsessive passion (OP). HP originates from an autonomous internalisation of the activity in identity and leads people to choose to be engaged in the activity that they love. It is expected that HP leads to more adaptive outcomes. Conversely, OP originates from a controlled internalisation in identity that leads people to an uncontrollable urge to engage in an activity (6). A study conducted by Vallerand et al. (6) showed that a higher level of passion led to deliberate practice and better performance. However, both types of passions seemed to be important in this context (7).

In soccer, HP can be found in players who freely choose to participate, with no sense of obligation, while OP can be found in those who have intense feelings about the sport. However, when there is a drive comes from the internal pressure, a feeling that the person ‘has to’ train, play and so forth. Both types of passions can lead to high-performance outcomes (1, 5). Vallerand (6) reported that preliminary evidence reveals that HP may positively contribute to psychological adjustment through its impact on the positive situational affect. OP, on the other hand, does not contribute to psychological adjustment and may even distract from it through right persistence in ill-advised activities, such as gambling. In sports, athletes with OP can experience a higher risk of maladaptive consequences, such as exhaustion, overtraining, and burnout (5). In sports, a number of researches had indicated relevant links between Vallerand’s passion model (including HP and OP) and diverse life activities (6); mastery goals and performance-avoidance goals (6), deliberate practice, performance attainment and subjective well-being (HP positively and OP negatively) (8), coach-athlete relationship (HP positively and OP negatively) (9), and flow (the positive relationship between the flow state and HP, and negative relationship between the flow state and OP). Furthermore, Vallerand’s passion model is also connected to intrinsic-extrinsic motivation (positively correlated with both HP and OP), amotivation (negative correlation with OP but positive correlation with HP), task and ego orientation (OP positively associated with both task orientation and ego orientation) and sports experience (less experienced athletes of 1 to 10 years, have a higher passion for sports activity compared to more experienced athletes over 11 years) (10). These findings revealed that passion matters not only in relation to intrapersonal outcomes (e.g. cognition, affect, psychological well-being, physical health and performance), but also for interpersonal, intergroup and societal consequences. These studies also address the importance of considering the nature of a situation to better predict the consequences of the types of passion in sports (6).

In sports, courage is frequently considered the principal virtue (11, 12). It is universally valued across cultures (13). Despite, there are increasing interest in understanding the concept of courage in sports (14), it has received little scientific attention (11). Thus, Konter and Ng (12) examined the initial development of specific multidimensional sports courage model leading to the development of the sports courage scale-31 (SCS-31) to measure courage (12). There are five subscales in courage, known as mastery (MT), determination (DT), assertiveness (AT), venturesome (VS) and sacrificial behaviour (SB).

Recently, Konter and Beckman’s (15) reviewed the importance of courage in sports and reported that courage is frequently considered a significant factor in sports performance. The researchers addressed courage from a self-regulation perspective. They indicated that courage in soccer should allow players to initiate actions and persistence in the pursuit of goals despite the risk. Thus, courage could act as a self-regulation process refers to the ability to face danger, difficulty, uncertainty, or pain and overcome the fear to maintain the chosen course of action.

Some soccer courage research using the SCS-31 approach indicated significant positive relationship in term of i) coping with stress, confidence and achievement motivation (16, 17); ii) commitment, peaking under pressure and imagery (18); iii) adventurous behaviour, perception of success, unaffectedness by negative referee decisions (19); iv) confidence building, unaffectedness by hostile and supportive spectators (20); v) team captains, proneness to injury, receiving more yellow and red cards, fewer chances of being selected for the national team (21); vi) higher risk-taking and injury proneness (15); and vii) problematic relationships with their coaches and managers (22).

Thus, research findings indicated that the concepts of soccer courage and soccer psychological skills seem to be related. It could be concluded that the positive relationship of better performance and courage in football is mediated by adventurous behaviour, perception of success and reduced irritability, which are components of courage (15). One of the characteristics of courageous players is that to a large degree, they do not conform but stand up for their convictions, even if that means opposition to the coach. Thus, the coach might perceive them as being quarrelsome and hard to coach. There is currently a limited understanding of the importance of the psychological aspect of courage in coaches and officials. In general, some coaches might demand that their players play it safe, stick to the game plan, tactics, and strategies, and carry out the positional demands assigned to them. This conflicts with the courageous player’s basic orientation that is driven by a high degree of autonomy. If they are forced to stick to the coach’s directions, it might interfere with their instinct to take risks and be creative in the game (15).

Courageous players are especially needed when the going gets tough. As the concept of courage suggests, more courageous footballers (with higher MT and AT) appear to be positively motivated by negative events (such as bad referee decisions or being behind at half time). Therefore, courage is needed under negative or aversive conditions to maintain or increase performance. A courageous leader on the pitch may be required to prevent the team from collapsing and turn the game around (23). However, the exact nature of the relationship between passion and courage in soccer performance is still relatively unknown. Therefore, the purpose of this study is to examine the passion possessed by male soccer players in relation to the level of courage, individual criteria (age, body mass index [BMI], years of experience, formal education and playing position), and performance variables.

## Methods

### Study Design, Sampling Method and Participants

Data collection was held in soccer training premises in Turkey. A cross-sectional study design with convenience sampling method was carried out among soccer players. The soccer players needed to have a minimum experience of one year in competitive soccer playing and were identified by their coaches. Then, the researchers approached the soccer players to be recruited in the study. The data collection was conducted between February and April 2019. All participants volunteered to participate in this study.

### Measures

The soccer players responded to the validated PS (24) and SCS-31 (12), together with their demographic and player characteristics.

#### Demographic form

The demographic form, which includes individual’s characteristics (age, BMI, years of competitive experience and playing position), and participants’ performance (the number years of representation at the national team, performance response in adverse decisions, performance response against weaker and stronger opponents, and level of participation) were collected.

#### PS

The original PS consisted of two six-item subscales assessing HP and OP. The PS is in Turkish language and has been validated in the Turkey population (24). It is a 7-point Likert scale ranging from 1 (do not agree at all) to 7 (completely agree). The internal consistency coefficients were 0.83 for HP and 0.78 for OP (24).

#### SCS-31

Konter and Ng (12) initially developed the SCS-31 using the large sample of athletes from individual and team sports (*n* = 768). The SCS-31 is in Turkish language and has been validated in Turkey population (12). Exploratory factor analyses (EFA) and confırmatory factor analyses (CFA) revealed a 5-factors structure of the SCS-31 that supported factorial validity and reliability of the scale. These factors were labelled as MT, DT, AT, VS and SB. EFA and CFA suggested a good fit: χ^2^(429) = 584.32, *P* < 0.01, comparative fit index (CFI) = 0.93, Tucker–Lewis index (TLI) = 0.93, root mean square error of approximation (RMSEA) = 0.03, standardised root mean residual (SRMR) = 0.06. The Cronbach alpha for the subscales were: MT = 0.82, DT = 0.82, AT = 0.72, VS = 0.72 and SB = 0.61. Then, test-retest reliability of the SCS-31 also supported strong reliabity, based on the responses from 75 athletes: MT = 0.77, DT = 0.73, AT = 0.67, VS = 0.74, SB = 0.62, total SCS-31 = 0.82 (12).

### Procedure

The study received approval from the first author’s institutional Human Research Ethics Committee and was conducted in accordance with the Declaration of Helsinki. Data were collected using convenience sampling with voluntary participation. Managers and coaches of the soccer clubs were contacted, and the research project explained. After the authorities granted relevant permissions, the time and place for the data collection were determined. Forms were distributed to the soccer players at their soccer training premises. Participants were encouraged to answer honestly and ensured confidentiality. The data collection took approximately 20 min to complete the set of forms.

### Statistical Analysis

Data analysis was conducted using the Statistical Package for the Social Sciences (SPSS version 25.0). Numerical variables were described as mean (M) ± standard deviation (SD), categorical variables were described as frequency (*n*) and percentage (%). The distributions of all numerical variables were assessed for normality by using histogram and Kolmogorov-Smirnov test. The numerical variables were or approximately to normal distribution; therefore, the collected data were analysed by one-way analysis of variance (ANOVA) and independent *t*-test. If one-way ANOVA of *F*-statistics test is significant, Bonferroni multiple pairs comparison test was used to local the differences. Assumption of equal variance in independent *t*-test and one-way ANOVA were checked by using Levene’s test.

### Sample Size

Sample size calculation was conducted by using GPower 3.1.9.7. Due to the lack of published study on passion and courage in sport, especially in soccer, therefore we estimate the sample size based on an effect size suggested by a psychologist expert in soccer. As this study is relatively new in the sport of soccer, thus small to medium effect size was suggested by the expert. We believed that the difference of passions’ score between low and high levels of courage would have an effect size of 0.35. Thus, with an alpha value of 0.05, power of 0.80, the sample size needed was 260 (130 per group). After adding 10% of dropout rate, a total sample size of 289 was needed. In the present study, total participants were 278, which fulfilled the minimum sample of 260.

## Results

### Participants’ Demographic Information

A total of 278 male soccer players aged ranging from 13–38 years (M = 17.42) (SD = 4.36); 233 amateurs, 43 professionals and two did not state their status) with the competitive soccer experiences ranging from 1–28 years (M = 7.51) (SD = 4.23), participated in the study. Table 1 shows the descriptive characteristics of the participants in this study. The mean age of participants was 17 years old and all participants had an average of eight years of soccer experiences.

### Levels of HP and Related Variables

Table 2 shows the differences levels of HP among different courage subscales’ groups (i.e. low, high). Soccer players with a higher level of MT, DT, AT, VS and total courage had significantly higher points of HP than soccer players with the lower level. Besides, there were significant differences between the levels of HP on experiences in soccer playing and soccer level. Professional soccer players have significantly higher points of HP than amateur soccer players.

### Levels of OP and Related Variables

Table 3 shows the differences levels of OP among different courage subscales’ groups (i.e. low, high). Soccer players with a higher level of AT and VS had significantly higher points of OP than soccer players with the lower level. Soccer players with experiences in soccer-playing (more than 10 years) and were professional players have significantly higher points of OP than those with less than 10 years of experiences and were amateur players, respectively. There was also a significant difference in OP among the three education levels. Interestingly, soccer players with secondary education background had higher points of OP than those with the university background.

### Performance Against Stronger Opponent

Table 4 shows the mean comparison of total courage score between different levels of performance against the stronger opponents (better, worse and no difference). There was significantly different between the mean of MT among the three performance categories. Soccer players with a better performance against stronger opponents have significantly higher points of MT than soccer players with worse performance.

### National Team Participation

Selected national soccer players have higher points of DT, AT, VS and total courage than nonselected national soccer players, however, the mean differences are not statistically significant (Table 5).

## Discussion

### HP

The results indicated that soccer players with higher points of sports courage factors (MT, DT, AT, VS and total courage) had significantly higher scores of HP than soccer players with lower points of sports courage. This could be important for the development of courage in terms of HP. The soccer player without courage could not generate the desired levels of HP and performance results. Therefore, passionate play is not sufficient for successful performance outcomes. For example, Holt and Dunn (25) studied successful youth elite players at an academy level or playing for their country’s youth national team. All the players referred to their love of the game as their primary motivation. However, in another study, players of the same age who were about to be cut from their club after being labelled unsuccessful, have the same motive and the love of the game as their core motive (26). In other words, both successful and unsuccessful players claimed that they have a similar motive. Therefore, motivation or HP without courage could not generate the desired results. For example, research indicated that characteristics that differentiate those who reach the professional level from those who do not include courage (15). Researchers also indicated some other factors could be related to HP, for example, psychological skills (27), mental toughness (28), coping (29), goal-setting, leadership, responsibility and emotional regulation (30), mastery climate (31), mindfulness (32), flow (10), coach-athlete relationship (9, 33), DT, goal commitment and strive for success, high levels of effort, invest much energy, and mobilising all possible resources to get where they want to be (1). However, the results of the present research showed that soccer courage is a major factor for HP. There is not enough HP research concerning sports courage to generalise indicated results. Future research could include these variables in relation to HP. More research is needed in order to provide conclusive results.

Besides, this also seems to be the case in relation to the years of experience as a function of age, whereby soccer players who have 1–10 years of experience have significantly higher points of HP than soccer players with 10 years of experience and above. This result is supported by Kelecek (24), who stated that professional soccer players have significantly higher HP points than amateur soccer players. Perception of the reward system could influence the motivation and HP of the soccer players. As a result, performance problems could arise. Therefore, more research is needed for more conclusive results in relation to age, years of experiences and level of soccer.

Results showed that the performance of soccer players with higher HP could either be better or worse than players with no performance difference after unfavourable decisions in soccer. Therefore, performance after unfavourable decisions could be more related to other psychological factors than HP (for example, courage). In other words, having performance consistency does not mean having performance deterioration under aversive and disadvantages conditions. Factors such as, DT and goal directed behaviour (1), MT goals (5, 6), 5C traits (i.e. commitment, communication, concentration, confidence and control) (34), anxiety (35), courage (27) could be important. Besides, HP seems to be more related to the performance and accuracy of affective forecast (36) than OP (5). High intrinsic involvement in soccer seems to be linked with passionate participation (1).

Jordet (1) suggested that coaches can stimulate intrinsic, self-determined motivation and HP in their players by i) providing an autonomy-supportive environment; ii) allowing players’ motivational needs to be met in relation to autonomy, relatedness and competence; iii) taking players’ perspectives, desires, feelings and choices seriously; and iv) establishing safe and genuinely sound relationships with players by demonstrating concern for them and by being approachable and trustworthy. However, the present research gives partial support for these contentions and HP in terms of performance. Results showed that courage appears to be more related to performance than HP and OP. More research is needed for more conclusive results.

### OP

The results indicated that soccer players with higher points of AT and VS have significantly higher scores of OP compared to soccer players with lower points of AT and VS. Therefore, similar comments could be made for OP as HP. Courage involves OP to the point, but it is a different concept from OP. However, we found not much research related to OP and soccer courage to generalise the results.

It appears that HP is slightly more correlated with soccer courage factors, including MT, DT, AT, VS, SB and total courage. It seems that VS and SB were more correlated with HP than OP. There is not enough research related to soccer courage, OP, and HP to make generalisations. The negative side of courage could be more related to OP, and the positive side of courage could be more related to HP as a self-regulatory and multidimensional concept (15). Extensive research is needed for more conclusive results.

Soccer players with ten years above of experience have significantly higher points of OP than soccer players with 10 years below. Similarly, professional soccer players had significantly higher points of OP than amateur soccer players. In addition, soccer players with secondary school education had significantly higher points of OP than soccer players with university education. All these results are similar to the HP results. Therefore, we can make the same or similar comments as indicated above with the HP.

### Sports Courage

Soccer players with a better performance against stronger opponents have significantly higher points of MT compared to soccer players with worse performance. It appears that courage is essential to challenge against superior opponents, as shown by the results of MT. The level of soccer courage could predict performance against stronger opponents. Therefore, soccer courage education could be an essential area that sports psychologist, coaches and club managers should consider. Results indicated that selected national soccer players had generally higher points of DT, AT and total courage than nonselected national soccer players. It seems that DT and AT play major roles to be selected for the national soccer teams. To increase players DT and AT could help their soccer performance and their selection for the national teams.

Some limitations should be noted, such as self-reporting nature of the surveys during the training sessions. Thus, some athletes might over-report or under-report some results. Besides, some soccer players missed some answers, such as their age or soccer experiences. In addition, not all soccer players volunteered to participate in this study. This study was also limited to male soccer players; hence results were limited to one gender. Future study should include female soccer players to examine their sports courage and passion for sports. With that said, the present study was based on a real-world study occurring in a real-world setting.

## Conclusion

In conclusion, the study revealed a number of significant differences between the passion scale scores of the soccer players and the level of courage (low and high), and their various individual and performance variables. Soccer players with higher courage had generally higher points of HP and OP. The courage and passion traits of the soccer players seemed to have an essential role in indicated individual and performance variables.

## Figures and Tables

**Table 1:** Descriptive characteristics of participant’s sociodemographic

Variables	M (SD)	*n* (%)
Age	17.42 (4.36)	271 (97.5)
Unstated		7 (2.5)
Soccer experiences (year)	7.51 (4.22)	263 (94.6)
Unstated		15 (5.4)
BMI	20.81 (2.58)	271 (97.5)
Unstated		7 (2.5)
National team participation (years)		
Non-selected national		258 (92.8)
Selected national		10 (3.6)
Unstated		10 (3.6)
Level of participation		
Amateur		233 (83.8)
Professional		43 (15.5)
Unstated		2 (0.7)
Playing position		
Goalkeeper		33 (11.9)
Full back		59 (21.2)
Stopper		26 (9.4)
Libero		10 (3.6)
Centre midfield		40 (14.4)
Right-left winger		51 (18.3)
Right-left midfield		26 (9.4)
Centre forward		30 (10.8)
Unstated		3 (1.1)
Passion		
HP	29.42 (6.18)	
OP	23.73 (6.78)	
Courage		
MT	20.87 (5.79)	
Low		146 (52.4)
High		132 (47.5)
DT	33.45 (5.84)	
Low		150 (54.0)
High		128 (46.9)
AT	24.09 (4.22)	
Low		152 (54.7)
High		126 (45.3)
VS	13.46 (2.74)	
Low		149 (53.6)
High		129 (46.4)
SB	12.94 (2.73)	
Low		152 (54.7)
High		126 (45.3)
Total courage	104.81 (15.98)	
Low		139 (50.0)
High		139 (50.0)

**Table 2:** HP in relation to related variables

Variables	M (SD)	MD (95% CI)	Test statistics (df)	*P*-value
MT		−2.82 (−3.51, −0.62)	−2.82 (276)*^t^*	0.005
Low	28.44 (6.06)			
High	30.50 (6.15)			
DT		−2.76 (−4.19, −1.33)	−3.80 (276)*^t^*	< 0.001
Low	28.15 (6.18)			
High	30.91 (5.86)			
AT		−2.26 (−3.70, −0.82)	−3.08 (276)*^t^*	0.002
Low	28.40 (5.94)			
High	30.67 (6.26)			
VS		−2.01 (−3.46, −0.57)	−2.74 (276)*^t^*	0.007
Low	28.49 (5.78)			
High	30.50 (6.46)			
SB		−1.44 (−2.89, −0.02)	−1.94 (276)*^t^*	0.054
Low	28.77 (5.92)			
High	30.21 (6.40)			
Total courage		−3.32 (−4.73, −1.92)	−4.65 (276)*^t^*	< 0.001
Low	27.76 (6.02)			
High	31.08 (5.90)			
Experience		2.08 (0.25, 3.93)	2.23 (261)*^t^*	0.026
1–10 years	29.97 (5.61)			
> 10 years	27.87 (6.85)			
Soccer level		−3.17 (−5.16, −1.18)	−3.14 (274)*^t^*	0.002
Amateur	26.71 (7.24)			
Professional	29.88 (5.86)			
Performance after unfavourable decision		–	2.86 (2, 273)*^f^*	0.059
Better	30.50 (6.55)			
Worse	29.85 (5.28)			
No difference	28.52 (6.23)			

Notes: *t* = *t*-statistic from independent *t*-test applied; *f* = *F*-statistic from one-way ANOVA test applied; MD = mean difference; CI = confidence interval

**Table 3:** OP in relation to related variables

Variables	M (SD)	MD (95% CI)	Test statistics (df)	*P*-value
MT		0.53 (−1.08, 2.13)	0.65 (276)*^t^*	0.520
Low	23.98 (7.06)			
High	23.45 (6.46)			
DT		−1.04 (−2.65, 0.56)	−1.28 (276)*^t^*	0.202
Low	23.25 (6.78)			
Hgh	24.29 (6.76)			
AT		−2.15 (−3.74, −0.56)	−2.67 (276)*^t^*	0.008
Low	22.75 (6.24)			
High	24.90 (7.22)			
VS		−1.96 (−3.55, −0.37)	−2.43 (276)*^t^*	0.016
Low	22.82 (6.53)			
High	24.78 (6.93)			
SB		−1.60 (−3.20, 0.01)	−1.97 (276)*^t^*	0.051
Low	23.00 (6.38)			
High	24.60 (7.16)			
Total courage		−1.16 (−2.76, 0.44)	−1.43 (276)*^t^*	0.154
Low	23.15 (6.78)			
High	24.31 (6.75)			
Experience		2.71 (0.63, 4.78)	2.57 (261)*^t^*	0.011
1–10 years	24.50 (6.35)			
> 10 years	21.80 (7.62)			< 0.001
Soccer level		−4.32 (−6.49, −2.16)	−3.93 (274)*^t^*	
Amateur	20.05 (5.91)			
Professional	24.38 (6.57)			
Performanc after unfavourable decision		–	0.149 (2, 273)*^f^*	0.861
Better	23.42 (7.37)			
Worse	24.00 (6.46)			
No difference	23.82 (6.61)			

Notes: *t = t*-statistic from independent *t*-test applied; *f = F*-statistic from one-way ANOVA test applied; MD = mean difference; CI = confidence interval

**Table 4:** Results of courage and performance against stronger opponent

Courage variables	Against stronger opponent categories	M (SD)	F-statistics (df)	P-value
MT	Performance		4.90 (2, 274)	0.008*
	1-Better	21.46 (5.47)		
	2-Worse	17.31 (4.24)		
	3-No difference	20.57 (6.60)		
DT	Performance		1.48 (2, 274)	0.230
	1-Better	33.34 (5.95)		
	2-Worse	31.91 (4.52)		
	3-No difference	34.14 (5.58)		
AT	Performance		1.33 (2, 274)	0.267
	1-Better	24.28 (3.92)		
	2-Worse	22.75 (4.67)		
	3-No difference	24.25 (4.29)		
VS	Performance		0.124 (2, 274)	0.883
	1-Better	13.50 (2.61)		
	2-Worse	13.24 (2.58)		
	3-No difference	13.56 (2.81)		
SB	Performance		0.256 (2, 274)	0.774
	1-Better	12.90 (2.65)		
	2-Worse	12.83 (1.76)		
	3-No difference	13.13 (2.87)		
Total courage	Performance		2.22 (2, 274)	0.110
	1-Better	105.48 (15.12)		
	2-Worse	98.04 (11.93)		
	3-No difference	105.65 (17.27)		

Notes:

* Bonferroni multiple pairs comparison: Better versus worse (*P* = 0.007), Better versus no difference (*P* = 0.701), Worse versus no difference (*P* = 0.061)

**Table 5:** Results of courage and national team participation

Variables	Groups	M (SD)	MD (95% CI)	*t*-statistics (*df*)	*P*-value
MT	Performance		−0.31 (−3.46, 4.07)	0.16 (266)	0.873
	1-Non-national	20.98 (5.84)			
	2-National	20.67 (8.15)			
DT	Performance		−3.06 (−6.81, 0.68)	−1.61 (266)	0.108
	1-Non-National	33.27 (5.97)			
	2-National	36.33 (3.27)			
AT	Performance		−2.65 (−5.34, 0.04)	−1.94 (266)	0.053
	1-Non-national	23.92 (4.26)			
	2-National	26.57 (3.40)			
VS	Performance		−0.15 (−1.91, 1.60)	−0.17 (266)	0.863
	1-Non-national	13.45 (2.77)			
	2-National	13.60 (2.62)			
SB	Performance		0.04 (−1.70, 1.78)	0.04 (266)	0.968
	1-Non-national	12.94 (2.67)			
	2-National	12.90 (4.29)			
Total courage	Performance		−5.53 (−15.77, 4.72)	−1.06 (266)	0.289
	1-Non-national	104.55 (16.24)			
	2-National	110.08 (13.15)			

Notes: *t*-statistic from independent *t*-test applied; MD = mean difference; CI = confidence interval

## References

[1] Jordet G, Strudwick T (2016). Psychology of elite soccer players. Soccer science.

[2] Deci EL, Ryan RM (1985). Intrinsic motivation and self-dertermination in human behavior.

[3] Deci EL, Ryan RM (2000). The “what” and “why” of goal pursuits: Human needs and the self-DT of behavior. Psychol Inq.

[4] Pain M, Strudwick T (2016). Mental interventions. Soccer science.

[5] Vallerand RJ, Roberts GC, Tressure DC (2012). The dualistic model of in passion in sport and exercise. Advances in motivation in sport and exercise.

[6] Vallerand RJ, Mageau GA, Elliot AJ, Dumais A, Demers M, Rousseau F (2008). Passion and performance attainment in sport. Psychol Sport Exerc.

[7] Amiot CH, Vallerand RJ, Blanchard CM (2006). Passion and psychological adjustment: a test of the person-environment fit hypothesis. Pers Soc Psychol Bull.

[8] Vallerand JR, Salvy S, Mageau AG, Elliot JA, Denis DP, Grouzet FME (2007). On the role of passion in performance. J Personal.

[9] Lafrenière MK, Jowett S, Vallerand RJ, Donahue EG, Lormer R (2008). Passion in sport: on the quality of the coach–athlete relationship. J Sport Exerc Psychol.

[10] Kelecek S (2013). Investigate the role of passion in predicting the flow, sport motivation and goal orientation in athletes. Master’s diss.

[11] Corlett J, Hollowchak A (2002). Virtue lost: courage in sport. Philosophy in sport.

[12] Konter E, Ng J (2012). Development of sport courage scale. J Hum Kinet.

[13] Dahlsgaard K, Peterson C, Seligman MEP (2005). Shared virtue: the convergence of valued human strengths across culture and history. Rev Gen Psychol.

[14] Pury CLS, Lopez SJ, Lopez SJ, Snyder CR (2009). Courage.

[15] Konter E, Beckmann J, Konter E, Beckmann J, Loughead MT (2019). Courage in football. Football psychology: from theory to practice.

[16] Hidrus AB, Kuan G, Konter E, Kueh YC (2017). Relationship between courage and coping among football player in Kelantan.

[17] Smith ER, Schutz WR, Smoll LF, Ptacek TJ (1995). Development and validation of a multidimensional measure of sport-specific psychological skills: athletic coping skills ınventory-28. J Sport Exerc Psychol.

[18] Konter E (2017). Psychological skills of football players in relation to level of courage, individual and performance variables.

[19] Konter E (2015). Courage of association football players: decision-making at risk.

[20] Konter E (2015). Courage and football performance in the face of negative and positive circumstances.

[21] Konter E (2015). Courage profile of adolescent football players in relation to their selected individual variables.

[22] Konter E (2015). Courage profile of professional footballers in relation to selected individual, social and performance variables.

[23] Apitzsch E, Konter E, Beckmann J, Loughead MT (2019). Collective collapse in football. Football psychology: from theory to practice.

[24] Kelecek S, Aşçı H (2013). Validity and reliability of “Passion Scale” for Turkish college athletes. J Sport Sci.

[25] Holt NL, Dunn JGH (2004). Towards a grounded theory of the psychosocial competencies and environmental conditions associated with soccer success. J Appl Sport Psychol.

[26] Holt NL, Michell T (2006). Talent development in English professional soccer. Int J Sport Psychol.

[27] Konter E, Beckmann J, Mallett C, Konter E, Beckmann J, Loughead MT (2019). Psychological skills for football players. Football psychology: from theory to practice.

[28] Coulter JT, Thelwell CR, Konter E, Beckmann J, Loughead MT (2019). Courage in football. Football psychology: from theory to practice.

[29] Schellenberg BJI, Gaudreau P, Crocker PRE (2013). Passion and coping: relationships with changes in burnout and goal attainment in collegiate volleyball players. J Sport Exerc Psychol.

[30] Taylor IM, Bruner MW (2012). The social environment and developmental experiences in elite youth soccer. Psychol Sport Exerc.

[31] Deighan M (2018). Exploring the relationship of passion and goal orientation with university athletes’ university experiences. A thesis submitted in partial fulfillment of the requirements of the honors program.

[32] Amemiya R, Sakairi Y (2019). The effects of passion and mindfulness on the intrinsic motivation of Japanese athletes. Pers Individ Dif.

[33] Jowett S, Lafrenière MK, Vallerand RJ (2012). Passion for activities and relationship quality: a dyadic approach. J Soc Pers Relat.

[34] Steptoe K, King T, Harwood C, Konter E, Beckmann J, Loughead MT (2019). The consistent psycho-social development of young football players: implementing the 5C’s as a whicle for interdisciplinary cohesion. Football psychology: from theory to practice.

[35] Nesti M, Strudwick T (2016). Performance mind-set. Soccer science.

[36] Verner-Filion J, Lafrenière MK, Vallerand RJ (2012). On the accuracy of affective forecasting: the moderating role of passion. Pers Individ Dif.

